# Incident urothelial cancer in the Malmö Diet and Cancer Study: cohort characteristics and further validation of ezrin as a prognostic biomarker

**DOI:** 10.1186/s13000-014-0189-5

**Published:** 2014-10-03

**Authors:** Christoffer Wennersten, Gustav Andersson, Karolina Boman, Björn Nodin, Alexander Gaber, Karin Jirström

**Affiliations:** Department of Clinical Sciences, Lund, Division of Oncology and Pathology, Lund University, Skåne University Hospital, 221 85 Lund, Sweden

**Keywords:** Malmö Diet and Cancer, Ezrin, Urothelial cancer, Prognosis

## Abstract

**Background:**

Reduced membranous expression of the cytoskeleton-associated protein ezrin has previously been demonstrated to correlate with poor prognosis in urothelial bladder cancer in several independent studies. The present study provides a first description of clinicopathological characteristics of incident urothelial cancers, not only located to the bladder, in the prospective, population-based cohort study Malmö Diet and Cancer. In addition, the prognostic value of ezrin expression is validated in primary tumours, and the longitudinal expression of ezrin examined in a subset of primary and recurrent tumours (n = 28).

**Methods:**

Among a total number of 355 incident tumours registered up until Dec 31 2010, 335 were located to the bladder. Immunohistochemical expression of cytoplasmic and membranous ezrin was evaluated in tissue microarrays with primary tumours from 272 cases and recurrent tumours from 28 cases. A combined score of the minimum, mean and maximum fraction and percentage of staining was calculated. Classification regression tree analysis was applied for selection of prognostic cutoff. Kaplan-Meier analysis, log rank test, univariable and multivariable Cox regression proportional hazards’ modeling were used to evaluate the impact of ezrin expression on 5-year overall survival (OS).

**Results:**

Ezrin expression could be evaluated in 263/272 primary and all 28 recurrent tumours. Membranous but not cytoplasmic ezrin was significantly reduced in recurrent compared to primary tumours (p < 0.001). Low cytoplasmic and membranous ezrin expression were associated with more advanced T-stage (p = 0.004, p < 0.001) and high-grade tumours (p = 0.025, p < 0.001), but not with age, sex, tumour location or smoking status. Both low cytoplasmic and membranous ezrin staining were associated with a significantly reduced 5-year OS (HR = 1.65; 95% CI 1.06-2.57 and HR = 2.51, 95% CI 1.52-4.17), but only low membranous ezrin remained prognostic after adjustment for age, sex, stage, grade and smoking status (HR = 1.69, 95% CI 1.00-2.85).

**Conclusions:**

This study provides a first description of the clinicopathological characteristics of 355 incident urothelial cancers in the Malmö Diet and Cancer Study up until 2010. In addition, the value of ezrin expression as a prognostic biomarker is further consolidated in this type of cancer.

**Virtual Slides:**

The virtual slide(s) for this article can be found here: http://www.diagnosticpathology.diagnomx.eu/vs/13000_2014_189

**Electronic supplementary material:**

The online version of this article (doi:10.1186/s13000-014-0189-5) contains supplementary material, which is available to authorized users.

## Background

Urothelial carcinoma (UC) or transitional cell carcinoma may arise anywhere in the urothelial tract from the renal pelvis to the urethra, with the majority of cases occurring in the urinary bladder [1]. Urinary bladder cancer makes up for the fifth most common cancer diagnosis in developed countries [2]. Despite the higher prevalence in men, studies have demonstrated that women are at greater risk of presenting with more advanced disease and have a worse prognosis [3]. Tobacco smoking is the leading cause of urothelial cancer [4], and other risk factors are occupational exposure to e.g. aromatic amines and polycyclic aromatic hydrocarbons [5], and family history [6]. An influence of dietary factors, e.g. high fat intake, has also been suggested [7].

Non-muscle invasive tumours of the bladder, i.e. tumours in stage Ta, T1 and carcinoma in situ (CIS or Tis) make up for about 75% of the primary diagnoses and are commonly treated with transurethral resection of the bladder (TURB) and/or intravesical instillation of Bacillus Calmette-Guérin (BCG), with the purpose to treat existing tumour as well as to prevent recurrence and progression [8]. Nevertheless, recurrent disease is a frequent phenomenon and the risk of progression into muscle-invasive disease remains unpredictable [9,10]. Thus, there is a great need for better prognostic tools in order to identify high-risk categories of non-muscle invasive tumours, which should be treated with early radical cystectomy. Muscle-invasive tumours, e.g. in stage T2 and beyond, are treated with radical cystectomy, neoadjuvant chemotherapy or both [11]. The efficacy of available chemotherapeutic is however variable, and there is also a need for better treatment predictive biomarkers in order to improve response rates for patients with advanced bladder cancer.

Despite a plethora of proposed biomarkers, none have yet been implemented into clinical protocols for the management of urothelial cancer. In a first study from 2009, Palou et al. found that reduced membranous expression of the membrane-cytoskeletal linking protein ezrin was associated with adverse tumour characteristics and a worse prognosis in 92 patients with T1G3 urothelial cell carcinoma of the bladder [12]. Another recent study on 104 bladder cancer cases of varying stages and grades, loss of membranous ezrin was found to correlate with adverse tumour characteristics, but the prognostic impact was not evaluated [13]. The prognostic value of ezrin expression was however confirmed in a recent study by our group, encompassing two independent patient cohorts, n = 100 and n = 342, respectively [14].

The purpose of this study was to provide a first description of incident UC cases in the Malmö Diet and Cancer Study (MDCS) up until December 31 2010, and to further validate the expression, clinicopathological correlates and prognostic significance of ezrin in UC, not only limited to the bladder. Moreover, a longitudinal study of ezrin expression was performed in a small series (n = 28) of paired primary and recurrent UC samples.

## Methods

### Study group

Between 1991 and 1996, a total number of 28 098 individuals; 11 063 (39.4%) men and 17 035 (60.6%) women, between 44–74 years were enrolled in the prospective, population-based cohort study Malmö Diet and Cancer Study (MDCS), from a background population of 74 138 [15]. All participants completed the baseline examination, which included a questionnaire, anthropometric measurements and a dietary assessment. The questionnaire covered information on physical activity, use of tobacco and alcohol, heredity, socio-economic factors, education, occupation, previous and current disease and current medication. In addition, blood samples were collected and stored in −80°C. Follow-up is performed annually by record-linkage to national registries for cancer and cause of death.

**Figure 1:**
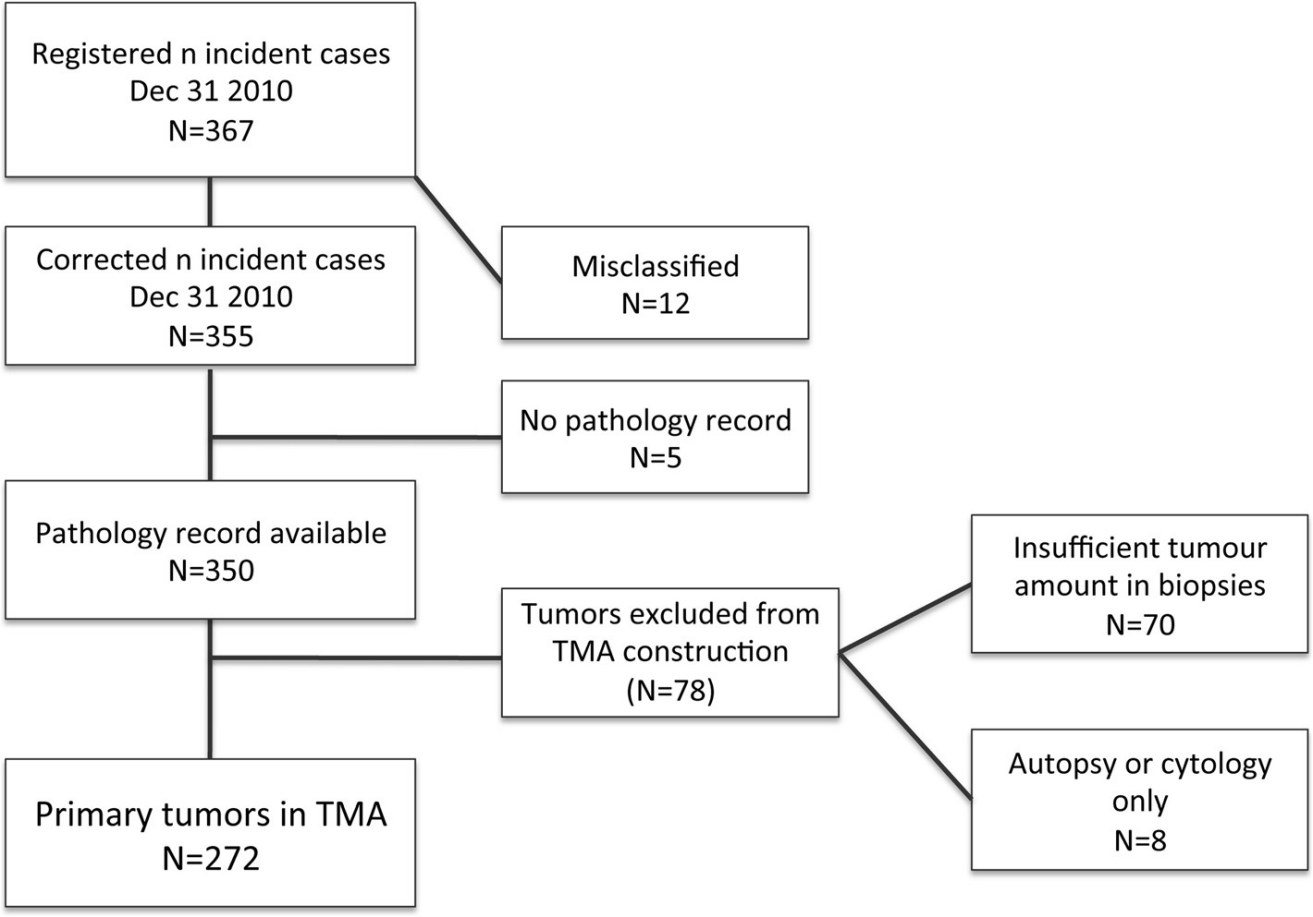
**Flowchart of urothelial cancers in the Malmö Diet and Cancer cohort.** A flowchart outlining the study cohort from a total number of 367 registered incident cases up until December 31^st^ 2010.

Until end of follow-up 31 December 2010, a total number of 367 incident cases of UC had been registered in the MDCS. All tumours were histopathologically re-evaluated and classified according to the WHO grading system of 2004 by a board certified pathologist. Twelve (3.3%) cases were misclassified, rendering a total number of 355 incident cases, of which 350 (98.6%) had available pathology records as well as archival specimens. Of the re-evaluated tumours, 78 (22.3%) cases were excluded from tissue microarray (TMA) construction due to an insufficient amount of tumour tissue (n = 70), or only cytology or autopsy specimens available (n = 8), rendering a total number of primary tumours from 272 cases eligible for TMA construction. A flowchart describing the cohort is provided in Figure 1.

Ethical permissions for the MDCS (Ref. 51/90), and the present study (Ref. 530/2008), were obtained from the Ethics Committee at Lund University.

### Tissue microarray construction

TMAs were constructed as previously described [14,16] using a semi-automated arraying device (TMArrayer, Pathology Devices, Westminister, MD, USA). For longitudinal analysis of biomarker expression, an additional TMA with matched samples from recurrent tumours was constructed from 28 cases, 21 (75.0%) of which were local, 4 (14.3%) regional (lymph node), and 3 (10.7%) distant recurrences. All recurrent tumours were chemonaive according to the pathology protocols. All primary and recurrent tumour samples were represented in duplicate tissue cores (1 mm).

### Immunohistochemistry and staining evaluation

For immunohistochemical analysis, 4 μm TMA-sections were automatically pre-treated using the PT Link system and then stained in an Autostainer Plus (DAKO; Glostrup, Copenhagen, Denmark) with a polyclonal, monospecific antibody (HPA021616, Atlas Antibodies AB, Stockholm, Sweden), diluted 1:1500. The specificity of the antibody has been confirmed by immunofluorescence, Western blotting and protein arrays (www.proteinatlas.org). Ezrin expression was annotated in accordance with previous studies [14], however using a further refined system. For evaluation of cytoplasmic staining, the percentage of cells with stained cytoplasm was denoted for each core, with a minimum value of 10% of a specific intensity needed in order to be accounted for, and also the proportion of cells with each degree of intensity (0–3), in percent. For example 10% cells with intensity 1, 40% with intensity 2 and 50% with intensity 3, were denoted as 0,1*1 + 0,4*2 + 0,5*3 for each given core. For evaluation of membranous ezrin, the percentage of cells with membranous staining was denoted (at least 5% for consideration), as well as the intensity of the staining (with a minimum proportion of 10% of a specific intensity required in order to be accounted for). The minimum, mean and maximum cytoplasmic and membranous ezrin expression was calculated from each tumour, based on their respective mean value of the annotated cores. The staining was evaluated by three independent observers, including one board certified pathologist, who were blinded to clinical and outcome data and every sample was re-evaluated once. Omission of primary antibody was used as a negative control, normal colonic mucosa as positive external control and lymphocytes as an internal positive control. Discrepant cases were re-evaluated once again and discussed in order to reach consensus.

### Statistical methods

Non-parametric Mann–Whitney U or Kruskal-Wallis tests were applied for analysis of the correlations between minimum, mean and maximum cytoplasmic and ezrin expression and clinicopathological characteristics. Wilcoxon-rank sum test was applied for comparison of ezrin expression in primary and recurrent tumours. Classification and regression tree (CRT) analysis was applied to assess optimal prognostic cut-offs for mean cytoplasmic and membranous ezrin expression. Receiver operating characteristics (ROC) curve analysis was also applied to verify the CRT-derived cutoffs. Kaplan-Meier analysis and log rank test were used to illustrate differences in five-year OS according to high and low ezrin expression. Cox regression proportional hazards modeling was used to estimate the impact patient and tumour characteristics on OS and of ezrin expression on five-year OS in both univariable and multivariable analysis, adjusted for age, sex, T-stage, grade and smoking history. All tests were two sided. P-values <0.05 was considered significant. All statistical analyses were performed using IBM SPSS Statistics version 20.0 (SPSS Inc., Chicago, IL, USA).

## Results

### Distribution of risk factors in cases and rest of cohort

The distribution of established risk factors of urothelial cancer for incident cases and rest of cohort is presented in Table 1. Age at baseline, the proportion of men and smokers was significantly higher in cases than in rest of cohort.

**Table 1:** **Distribution of established risk factors in urothelial cancer cases and rest of cohort**

**Characteristics**	**Rest of cohort**	**Cases**	***P***
n (%)	27743	355	
Age at baseline			<0.001
Mean, median	58.1, 57.7	61.7, 62.4	
(range)	(44.5-73.6)	(45.4-73.0)	
Sex			<0.001
Male	10817 (39.0)	246 (69.3)	
Female	16925 (61.0)	109 (30.7)	
Smoking habits			<0.001
regularly	6549 (23.6)	125 (35.2)	
occasionally	1239 (4.5)	23 (6.5)	
Former smoker	9351 (33.7)	155 (43.7)	
Never smoker	10592 (38.2)	52 (14.6)	
*missing*	*12*		

### Clinicopathological characteristics

Patient and tumour characteristics in the entire cohort of incident urothelial cancer, the TMA cohort and the non-TMA cohort are shown in Table 2. As expected, the vast majority of cases with known location (95.7%) were located to the bladder. The proportion of pTa tumours was higher in the non-TMA cohort, and pT1 tumours were more frequent in the TMA cohort. The non-TMA cohort included no tumours of stage T2 and above, and all CIS cases were excluded from the TMA-construction upfront, and therefore not included in the correlation analyses. Age, sex, tumour location and smoking habits did not differ between the TMA and the non-TMA cohort. The distribution of patients and tumour characteristics did not differ significantly between bladder (n = 335) and non-bladder (n = 15) tumours (data not shown), but, of note, there was a more equal distribution of sex among the latter with 7 (46.7%) females and 8 (53.3%) males compared to 100 (29.9%) females and 235 (70.1%) males in the former.

**Table 2:** **Patient and tumour characteristics in the entire incident cohort, and in cases with tumours included and excluded from tissue microarray construction, respectively**

**Factor**	**Entire cohort**	**TMA cohort**	**Non-TMA cohort**	***P***
n (%)	355 (100)	272 (76.6)	83 (23.4)	
**Age**				0.908
Mean, median	71.4, 71.7	71.4, 71.5	71.4, 72.2	
Range	51.2-86.6	51.2-86.8	56.3-84.2	
**Sex**				0.895
Women	109 (30.7)	84 (30.9)	25 (30.1)	
Men	246 (69.3)	188 (69.1)	58 (69.9)	
**T stage**				<0.001
pTa	163 (45.9)	116 (42.6)	47 (67.1)	^¶^<0.001
pT1	97 (27.3)	86 (31.6)	11 (15.7)	
pT2	64 (18.0)	64 (23.5)	0 (0.0)	
pT3	1 (0.3)	1 (0.4)	0 (0.0)	
pT4	5 (1.4)	5 (1.8)	0 (0.0)	
CIS	12 (3.5)	0 (0.0)	12 (17.1)	
*Missing*	*13*	*0*	*13*	
**Grade**				<0.001
Low	183 (51.5)	136 (50.0)	47 (77.0)	
High	150 (42.3)	136 (50.0)	14 (23.0)	
*Missing*	*22*	*0*	*22*	
**Location**				0.108
Bladder	335 (95.7)	264 (97.1)	71 (91.0)	
Urether	9 (2.6)	4 (1.4)	5 (6.4)	
Urethra	1 (0.3)	1 (0.4)	0 (0.0)	
Renal pelvis	5 (1.4)	3 (1.1)	2 (2.6)	
*Unknown*	*5*	*0*	*5*	
**Smoking habits**				0.851
Regularly	125 (35.2)	99 (36.4)	26 (31.3)	
Occasionally	23 (6.5)	15 (5.5)	8 (9.6)	
Former	155 (43.7)	116 (42.6)	39 (47.0)	
Never	52 (14.6)	42 (15.4)	10 (12.0)	

^¶^Significance level after exclusion of CIS.

The impact of clinicopathological factors on survival during follow-up until Dec 31st 2012 is shown in Table 3. In line with the expected, age at diagnosis, T-stage and grade were independent prognostic factors. The prognosis for cases with Tis did not differ significantly from pTa cases. Sex, tumour location and smoking habits were not associated with survival.

**Table 3:** **Risk of death during follow-up according to clinicopathological factors in the entire cohort**

**Factor**	**Unadjusted**		**Adjusted**	
	**n (events)**	**HR (95% CI)**	**n (events)**	**HR (95% CI)**
**Age**				
Continuous	355 (166)	1.06 (1.04-1.09)	342	1.05 (1.03-1.08)
**Gender**				
Female	109 (42)	1.00	103 (38)	1.00
Male	246 (124)	1.30 (0.92-1.85)	239 (122)	1.17 (0.81-1.70)
**Stage**				
Ta	163 (63)	1.00	163 (63)	1.00
T1	97 (42)	1.61 (1.09-2.39)	97 (42)	1.16 (0.73-1.82)
T2	64 (48)	3.97 (2.71-5.82)	64 (48)	2.60 (1.60-4.23)
T3-4	6 (5)	13.13 (5.16-33.39)	6 (5)	7.81 (2.99-20.41)
Tis	12 (2)	0.38 (0.09-1.56)	12 (2)	0.42 (0.10-1.71)
**Grade**				
Low	183 (65)	1.00	182 (64)	1.00
High	150 (96)	2.54 (1.85-3.48)	148 (94)	1.73 (1.14-2.62)
**Location**				
Bladder	335 (158)	1.00	327 (153)	1.00
Other	15 (7)	0.84 (0.39-1.79)	15 (7)	0.87 (0.38-1.95)
**Smoking**				
Regularly	125 (64)	1.00	120 (61)	1.00
Occasionally	23 (9)	0.63 (0.31-1.26)	23 (9)	0.69 (0.34-1.42)
Former	155 (71)	0.84 (0.60-1.18)	150 (71)	0.87 (0.61-1.25)
Never	52 (22)	0.81 (0.50-1.31)	49 (19)	0.86 (0.50-1.48)

### Distribution of ezrin expression in primary and metastatic tumours

**Figure 2:**
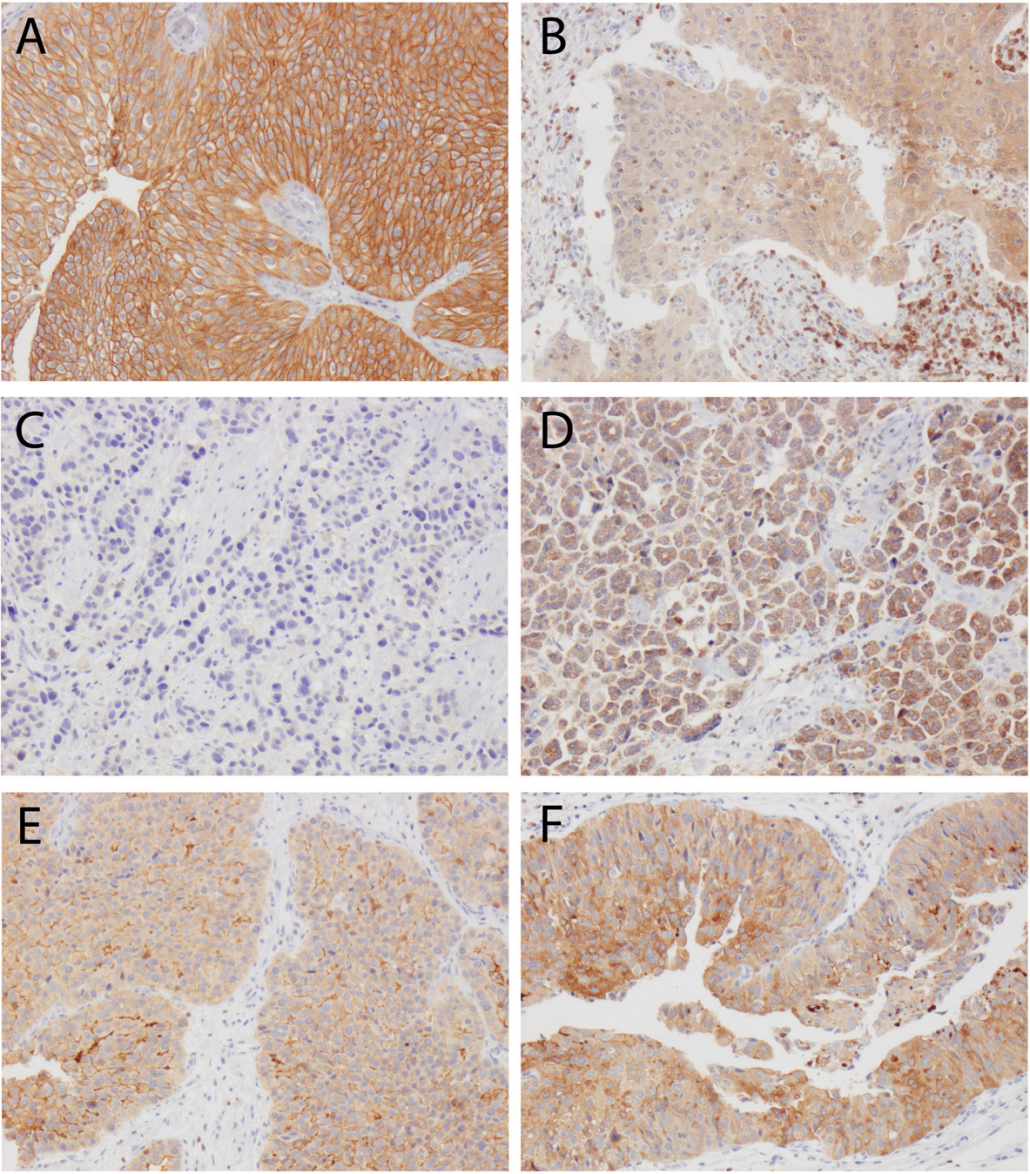
**Sample immunohistochemical images.** Images representing **A)** a primary tumour with strong membranous and moderate cytoplasmic ezrin expression in nearly 100% of cells, **B)** a primary tumour with negative membranous and weak, focally moderate cytoplasmic staining (note strongly staining stromal lymphocytes). **C)** A primary tumour with negative membranous and focally weak cytoplasmic ezrin expression and **D)** subsequent metastasis to the small intestine with negative membranous and moderate to strong cytoplasmic staining. **E)** A primary tumour with moderate to strong membranous ezrin expression in approximately 25% and weak cytoplasmic staining in the majority of cells and **F)** local recurrence with membranous ezrin expression in <5% and moderate to strong cytoplasmic ezrin expression in > 50% of cells.

Following antibody optimisation and staining, ezrin expression could be evaluated in 263/272 (96.7%) primary tumours and all 28 metastases. Lost cases were either a result of complete tissue loss during IHC preparation or an insufficient quantity of tumour tissue. Sample IHC images are shown in Figure 2.

**Figure 3:**
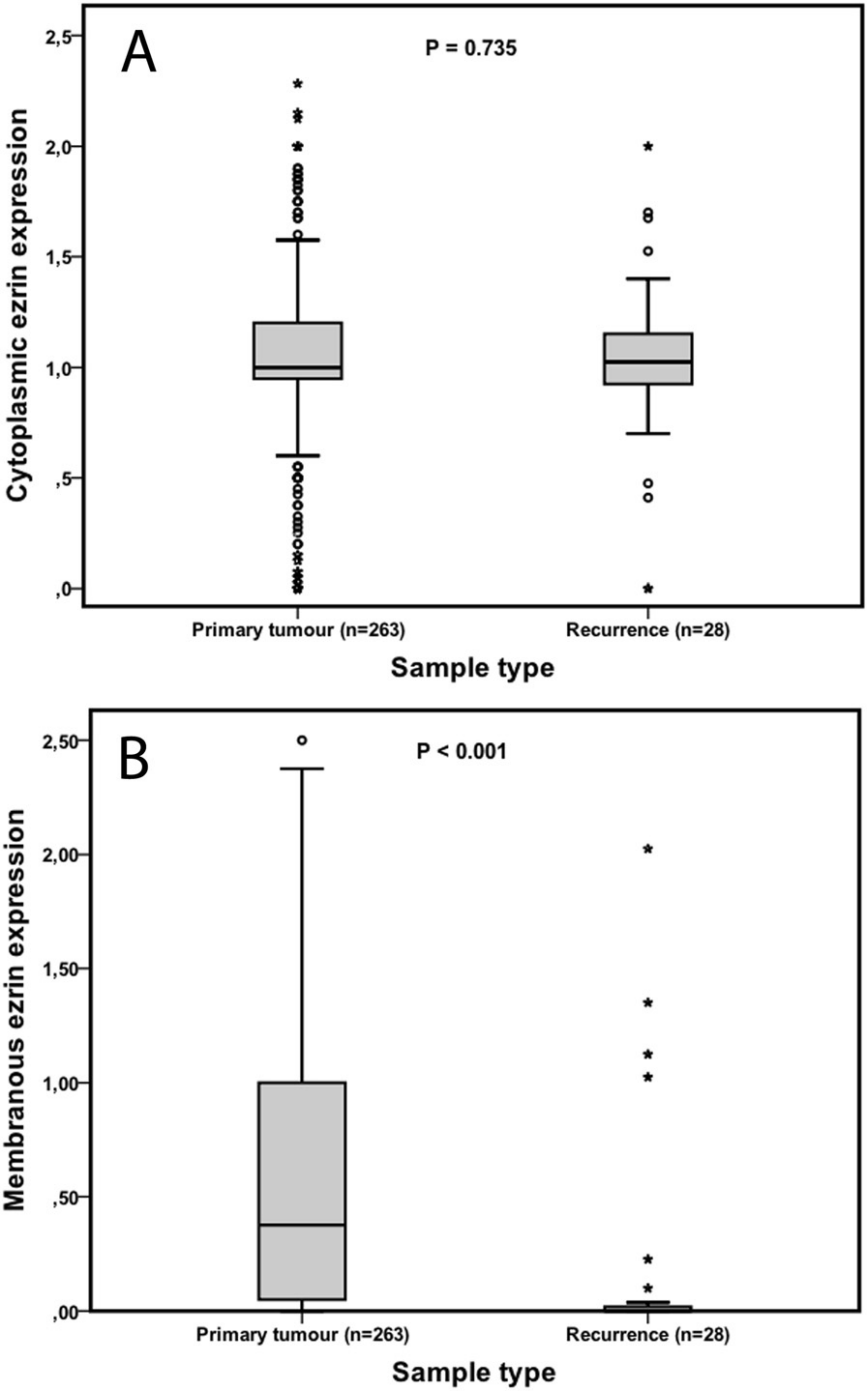
**Distribution of ezrin expression in primary and recurrent tumours.** Bar charts visualizing the distribution of mean **A)** cytoplasmic and **B)** membranous ezrin expression in primary and recurrent tumours.

As demonstrated in Figure 3, membranous (p < 0.001) but not cytoplasmic ezrin expression was demonstrated to be significantly downregulated in recurrent compared to primary tumours.

### Associations of ezrin expression with clinicopathological characteristics

As shown in Table 4, analyses of the relationship between cytoplasmic membranous staining and established clinicopathological factors revealed similar associations for minimum, mean and maximum cytoplasmic and membranous expression with clinicopathological factors. Significant associations were seen for both cytoplasmic and membranous ezrin expression with more advanced T-stage (p = 0.004 and p < 0.001, respectively, for mean expression) and high-grade tumours (p = 0.025 and p < 0.001, respectively, for mean expression), but not with age, sex, tumour location or smoking status.

**Table 4:** **Associations of cytoplasmic and membranous ezrin expression with clinicopathological parameters**

		**Min cytoplasmic**		**Mean cytoplasmic**		**Max cytoplasmic**		**Min membranous**		**Mean membranous**		**Max membranous**	
**Factor**	**n (%)**	**Mean, median (range)**	***P***	**Mean, median (range)**	***P***	**Mean, median (range)**	***P***	**Mean, median (range)**	***P***	**Mean, median (range)**	***P***	**Mean, median (range)**	***P***
**Age**													
≤ average	129	0.95, 1.00	*0.332*	1.03, 1.00	*0.77*	1.12, 1.00	*0.77*	0.46, 0.15	*0.234*	0.59, 0.37	*0.423*	*0.72, 0.67*	*0.680*
	(49.0)	(0.00-2.00)		(0.00-2.13)	*3*	(0.00-2.25)	*7*	(0.00-2.50)		(0.00-2.50)		(0.00-2.50)	
> average	134	0.90, 2.00		1.00, 1.00		1.12, 1.00		0.35, 0.10		0.52, 0.32		0.69, 0.50	
	(51.0)	(0.00-2.00)		(0.00-2.28)		(0.00-2.90)		(0.00-2.13)		(0.00-2.25)		(0.00-2.38)	
**Gender**													
Female	81	0.91, 1.00	*0.781*	1.00, 1.00	*0.76*	1.08, 1.00	*0.89*	0.46, 0.15	*0.348*	0.61, 0.62	*0.362*	0.77, 0.97	*0.576*
	(30.8)	(0.00-2.00)		(0.00-2.28)	*0*	(0.00-2.90)	*1*	(0.00-1.70)		(0.00-1.70)		(0.00-2.38)	
Male	182	0.92, 1.00		1.03, 1.00		1.13, 1.00		0.37, 0.10		0.53, 0.32		0.68, 0.51	
	(69.2)	(0.00-2.00)		(0.00-2.13)		(0.00-2.25)		(0.00-2.50)		(0.00-2.50)		(0.00-2.50)	
**T-stage**													
Ta	114	0.95, 1.00	*0.005*	1.05, 1.00	*0.00*	1.15, 1.00	*0.00*	0.50, 0.20	*0.001*	0.66, 0.59	*<0.001*	0.83, 0.90	*<0.001*
	(43.3)	(0.00-2.00)		(0.00-2.15)	*4*	(0.00-2.80)	*5*	(0.00-2.13)		(0.00-2.25)		(0.00-2.38)	
T1	84	0.99, 1.00		1.09, 1.05		1.19, 1.10		0.41, 0.15		0.62, 0.54		0.83, 0.72	
	(31.9)	(0.00-2.00)		(0.00-2.28)		(0.00-2.90)		(0.00-2.38)		(0.00-2.38)		(0.00-2.38)	
T2-4	65	0.78, 0.95		0.88, 0.97		0.97, 1.00		0.21, 0.05		0.27, 0.07		0.34, 0.10	
	(24.7)	(0.00-2.00)		(0.00-2.00)		(0.00-2.10)		(0.00-2.50)		(0.00-2.50)		(0.00-2.50)	
**Grade**													
Low	133	1.01, 1.00	*0.014*	1.10, 1.00	*0.02*	1.19, 1.00	*0.11*	0.49, 0.15	*0.008*	0.66, 0.60	*<0.001*	0.84, 0.97	*<0.001*
	(50.6)	(0.00-2.00)		(0.00-2.28)	*5*	(0.00-2.90)	*4*	(0.00-2.05)		(0.00-2.50)		(0.00-2.50)	
High	130	0.83, 1.00		0.94, 1.00		1.04, 1.00		0.31, 0.10)		0.44, 0.20		0.57, 0.20	
	(49.4)	(0.00-2.00)		(0.00-2.00)		(0.00-2.00)		(0.00-1.90)		(0.00-2.14)		(0.00-2.38)	
**Location**													
Bladder	258	0.93, 1.00	*0.934*	1.03, 1.00	*0.52*	1.13, 1.00	*0.51*	0.40, 0.10	*0.136*	0.56, 0.37	*0.040*	0.71, 0.52	*0.023*
	(98.1)	(0.00-2.00)		(0.00-2.28)	*9*	(0.00-2.90)	*0*	(0.00-2.50)		(0.00-2.50)		(0.00-2.50)	
Other	5	0.82, 1.00		0.83, 1.00		0.84, 1.00		0.24, 0.00		0.24, 0.00		0.24, 0.00	
	(1.9)	(0.00-1.10)		(0.00-1.10)		(0.00-1.10)		(0.00-1.20)		(0.00-1.20)		(0.00-1.20)	
**Smoking status**													
Regularly	93	0.98, 1.00	*0.384*	1.07, 1.00	*0.22*	1.17, 1.00	*0.15*	0.42, 0.10	*0.461*	0.58, 0.50	*0.300*	*0.75, 0.60*	*0.360*
	(35.4)	(0.00-2.00)		(0.00-2.13)	*3*	(0.00-2.25)	*6*	(0.00-2.50)		(0.00-2.50)		(0.00-2.50)	
Occasionally	15	0.89, 1.00		1.05, 1.00		1.21, 1.00		0.43, 0.20		0.57, 0.55		0,72, 0.90	
	(5.7)	(0.05-1.50)		(0.08-1.55)		(0.10-2.00)		(0.00-1.20)		(0.00-1.31)		(0.00-1.50)	
Former	113	0.85, 1.00		0.93, 1.00		1.01, 1.00		0.34, 0.10		0.49, 0.22		0.63, 0.35	
	(43.0)	(0.00-2.00)		(0.00-2.00)		(0.00-2.10)		(0.00-2.38)		(0.00-2.38)		(0.00-2.38)	
Never	42	1.00, 1.00		1.12, 1.02		1.24, 1.05		0.49, 0.17		0.65, 0.56		0.83, 0.75	
	(16.0)	(0.00-1.85)		(0.00-2.28)		(0.00-2.90)		(0.00-1.75)		(0.00-1.75)		(0.00-2.00)	

### Prognostic value of ezrin expression

CRT analysis determined optimal prognostic cut-offs at 0.988 for mean cytoplasmic and 0.538 for mean membranous ezrin expression in relation to 5-year OS (Additional file 1A and B). The same cutoffs were obtained for OS (data not shown), and by ROC analysis (data not shown).

**Figure 4:**
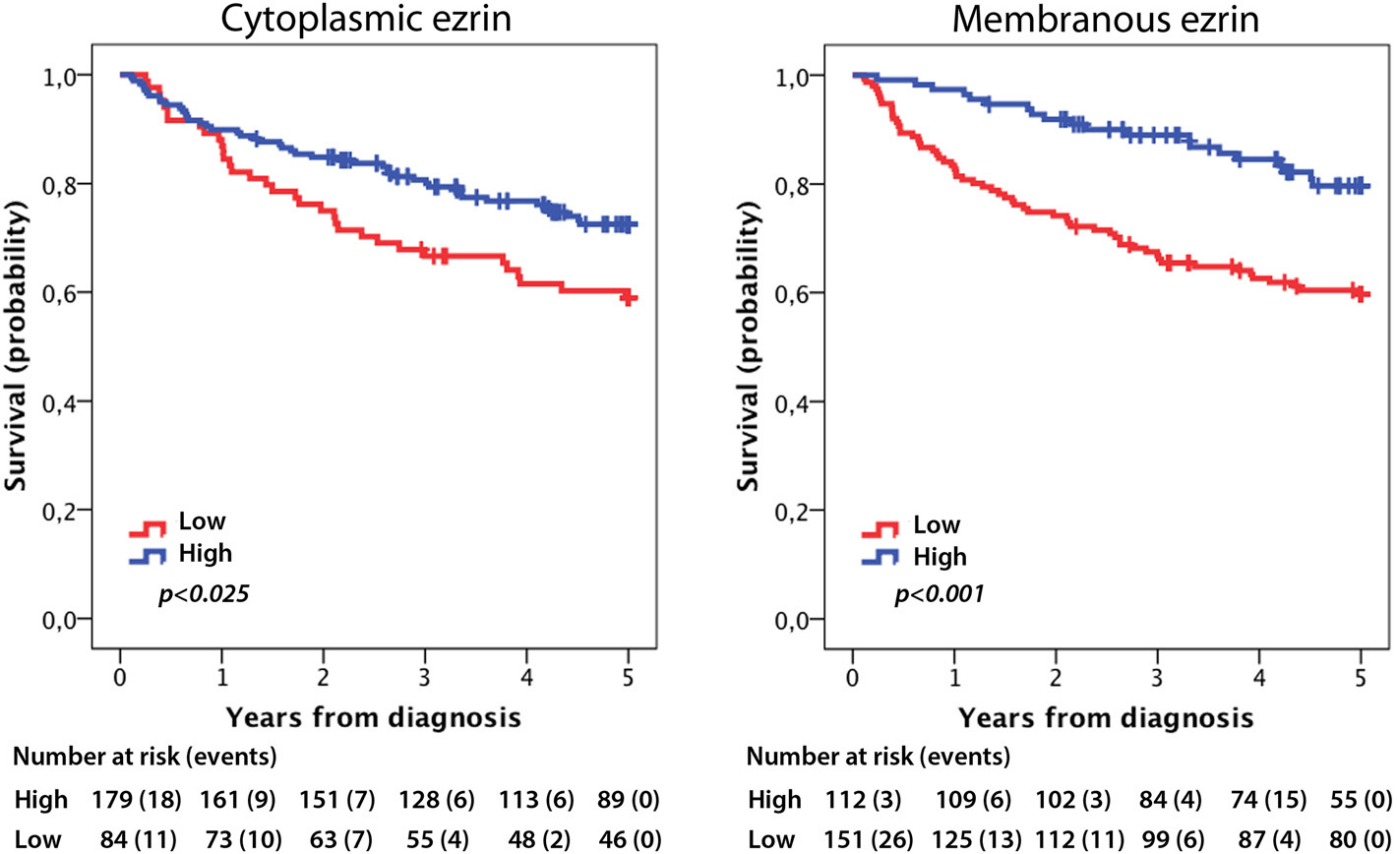
**Kaplan-Meier estimates of bladder cancer specific survival and 5-year overall survival.** Kaplan-Meier analysis of 5-year overall survival according to low and high cytoplasmic and membranous ezrin expression.

As demonstrated in Figure 4, low cytoplasmic (logrank p = 0.025) and membranous (logrank p < 0.001) ezrin expression were associated with a significantly shorter 5-year OS. For cytoplasmic ezrin, the association with survival was confirmed in univariable Cox regression analysis (HR = 1.65; 95% CI 1.06-2.57), but did not remain significant in multivariable analysis (HR = 1.11; 95% CI 0.70-1.77). As demonstrated in Table 5, the prognostic value of membranous ezrin was confirmed in both unadjusted (HR = 2.51, 95% CI 1.52-4.17) and adjusted (HR = 1.69, 95% CI 1.00-2.85), and these associations were slightly more significant when only bladder tumours were included in the analysis. In bladder tumours only, cytoplasmic ezrin expression was prognostic in unadjusted but not in adjusted analyses (data not shown). Neither cytoplasmic nor membranous ezrin expression was prognostic in non-invasive (pTa, pT1) tumours (data not shown). In tumours of pT2 stage and beyond cytoplasmic ezrin was not prognostic (data not shown), whereas loss of membranous ezrin was borderline prognostic (univariable and multivariable HR = 2.72, 95% CI 0.97-7.63).

**Table 5:** **Relative risks of death within 5 years according to clinicopathological factors and membranous ezrin expression in the entire cohort and in patients with bladder cancer**

	**Entire cohort**			**Bladder**		
		**Unadjusted**	**Adjusted**		**Unadjusted**	**Adjusted**
	**n (events)**	**HR (95% CI)**	**HR (95% CI)**	**n (events)**	**HR (95% CI)**	**HR (95% CI)**
**Age**						
Continuous	263 (80)	1.05 (1.01-1.08)	1.03 (0.999-1.07)	258 (80)	1.05 (1.01-1.08)	1.03 (0.998-1.07)
**Gender**						
Female	81 (23)	1.00	1.00	78 (23)	1.00	1.00
Male	182 (57)	1.10 (0.68-1.79)	0.94 (0.56-1.58)	180 (57)	1.06 (0.65-1.73)	0.91 (0.54-1.54)
**Stage**						
Ta	114 (15)	1.00	1.00	112 (15)	1.00	1,00
T1	84 (22)	2.40 (1.24-4.62)	1.86 (0.86-4.02)	81 (22)	2.47 (1.28-7.68)	2.38 (1.23-4.60)
T2-4	65 (43)	8.83 (4.88-15.97)	5.46 (2.41-12.36)	65 (43)	8.68 (4.80-15.70)	7.12 (3.87-13.08)
**Grade**						
Low	133 (20)	1.00	1.00	129 (20)	1.00	1.00
High	130 (60)	4.02 (2.43-6.68)	1.44 (0.71-2.92)	129 (60)	3.94 (2.37-6.54)	1.33 (0.65-2.73)
**Smoking**						
Regularly	93 (30)	1.00	1.00	92 (30)	1.00	1.00
Occasionally	15 (4)	0.76 (0.27-2.17)	0.90 (0.31-2.58)	15 (4)	0.75 (0.26-2.14)	0.90 (0.31-2.57)
Former	113 (32)	0.86 (0.52-1.42)	0.73 (0.44-1.21)	110 (32)	0.88 (0.53-1.44)	0.74 (0.45-1.23)
Never	42 (14)	1.04 (0.55-1.95)	1.22 (0.64-2.33)	41 (14)	1.05 (0.56-1.99)	1.26 (0.66-2.39)
**Ezrin**						
High	112 (20)	1.00	1.00	111 (20)	1.00	1.00
Low	151 (60)	2.51 (1.52-4.17)	1.69 (1.00-2.85)	147 (60)	2.58 (1.55-4.28)	1.74 (1.03-2.95)

Similar associations with prognosis were seen for minimum and maximum ezrin expression (data not shown).

## Discussion

The present study provides a first clinicopathological description of incident urothelial cancers in the Malmö Diet and Cancer Study up until 31^st^ December 2010, a total number of 355 tumours of which 272 have been assembled in TMAs to provide a long-lasting platform for studies into the molecular pathological epidemiology (MPE) of urothelial cancer. The term MPE refers to a multidisciplinary approach, initially proposed in 2010 [17], with the aim to investigate the relationship between various exposure factors with molecular characteristics of tumours, and particular progress has been made in research related to colorectal cancer [18,19]. However, since urothelial cancer is a disease that affects the entire urothelium and therefore particularly strongly associated with the term “field cancerization” [20,21], MPE research with an etiologically focused approach to the field effect will also be highly relevant in this type of cancer [22].

The distribution of patient and tumour characteristics in the here described cohort of incident urothelial cancers in the MDCS is in line with the expected. i.e. a predominance of male sex, non-invasive (pTa) tumours, and location to the bladder. The proportion of males and smokers was also significantly higher in cases compared with non-cases. Neither sex nor smoking status was however associated with overall survival, whereas both T-stage and tumour grade were strong prognostic factors. Information on nodal stage was not available, but since all tumours represented the first diagnosis, the portion of cases having metastatic disease upfront should be negligible.

This paper also provides a validation of the prognostic impact of ezrin expression in urothelial cancer. In line with several recent studies [12-14], loss of ezrin expression, in particular its membranous subcellular location, was found to be associated with tumours of more advanced T-stage and higher grade, and an independent predictor of a reduced 5-year overall survival. In this study, cytoplasmic ezrin expression was also found to be associated with adverse tumour characteristics and survival, although the prognostic value did not remain independent of conventional clinicopathological factors. This is in contrast to findings from our previous study [14] and another recent study [12], wherein cytoplasmic ezrin expression was not found to be associated with neither clinicopathological factors nor survival. Of note, in thepresent study, we used a more fine-tuned scoring system, wherein the minimum and maximum expression of ezrin was also taken into consideration. The clinicopathological correlates and prognostic impact were however similar for all variables, with the overall best correlations for the mean values. The application of automated approaches may add additional value to future validatory studies, in order to further determine optimal prognostic cutoffs for ezrin expression in urothelial carcinoma [23-25].

Of note, in contrast to previous studies related to the prognostic values of ezrin, the present study also included a small subset of non-bladder tumours. The distribution of patient and tumour characteristics did not differ from bladder tumours, but there was a more equal distribution of sexes, which is in line with previous findings [1]. Moreover, while the expression of ezrin did not differ significantly by tumour location, the prognostic impact of ezrin was slightly accentuated in the subgroup analysis of bladder tumours only. However, in light of the small number of cases with non-bladder tumours available for analysis, this observation may well be coincidental. The distribution of tumour stage and grade did not differ between bladder and non-bladder UC in the present cohort, but upper UC has been demonstrated to present with slighty more advanced tumour stage and grade in larger series [26,27]. Tumour location was however not prognostic, which is in line with findings from previous studies [28]. Nevertheless, it would inarguably be of interest to further examine the prognostic value of ezrin expression in a larger series of non-bladder urinary tract carcinoma.

Of note, only one case with non-bladder location was a urethral cancer, a T2G3 tumour with partly sarcomatoid differentiation in a male patient who died within three months from diagnosis. This tumour was not included in the TMA-analysis. Urethral cancer is a rare disease, with somewhat different aetiology and an often more aggressive clinical course than UC of the bladder and upper urothelial tract [29].

In this study, the prognostic value of ezrin was only examined against 5-year overall survival. Previous studies have however demonstrated an effect also on progression-free survival [12] but not on the recurrence rate [14]. Of note, in our previous paper, we did not find an association between loss of ezrin expression and risk of progression to muscle-invasive disease in pTa-pT1 tumours [14].

As a novel feature, the present paper also entails a longitudinal study of ezrin expression, e.g. comparison of expression in primary and recurrent tumours, the majority being local recurrences, in a small subset of cases. Despite the small sample size, the finding of a significant reduction of membranous ezrin expression in recurrent tumours further supports the increasing body of evidence demonstrating that loss of ezrin is associated with tumour progression. In contrast, there was no significant difference in cytoplasmic ezrin expression between primary and recurrent tumours. This finding may well be due to chance, given the small sample size, but is also in line with the observation of cytoplasmic ezrin seemingly being more weakly associated with aggressive tumour features. Nevertheless, future validatory studies on the prognostic value of ezrin expression in UC should consider both its cytoplasmic and membranous expression. The herein observed discrepancy between ezrin expression in primary and metastatic tumours also indicates that the metastases may be the most suitable tissue for biomarker studies on patients with clinically advanced UC. It will also be relevant to examine the effects of neoadjuvant chemotherapy on ezrin expression in tumours from cystectomy specimens, to determine their suitability for prognostication purposes [30].

The prognostic value of ezrin expression at the mRNA level has not yet been determined, but immunohistochemistry has several advantages in that it allows for assessment of biomarkers in a subcellular and histological context, and can easily be incorporated into clinical protocols. Morever, there may well be a prognostic disconcordance between mRNA and protein levels of candidate biomarkers [31].

## Conclusions

This study provides a first description of the clinicopathological characteristics of 355 incident urothelial cancers in the Malmö Diet and Cancer Study up until 2010, demonstrating the suitability of this cohort for molecular pathological epidemiology research related to these cancers. In addition, the value of ezrin expression as a prognostic biomarker in urothelial cancer has been further consolidated. Apart from an independent predictive value with regard to five-year overall survival, membranous ezrin expression was also demonstrated to be significantly reduced in recurrent disease.

### Consent

Written informed consent was obtained from each subject included in the study.

## Additional file

Additional file 1:
**Classification regression tree analysis for selection of prognostic cutoffs.** (A) Cytoplasmic and (B) membranous expression.

## Abbreviations

MDCS: Malmö Diet and Cancer Study
TMA: Tissue microarray
CRT: Classification regression tree
OS: Overall survival
HR: Hazard ratio
CI: Confidence interval
