# Supplementary figures and images for: Incident urothelial cancer in the Malmö Diet and Cancer Study: cohort characteristics and further validation of ezrin as a prognostic biomarker
Source: Diagn Pathol. 2014 Oct 3;9:189. doi: 10.1186/s13000-014-0189-5 (PMC4195979; doi:10.1186/s13000-014-0189-5)

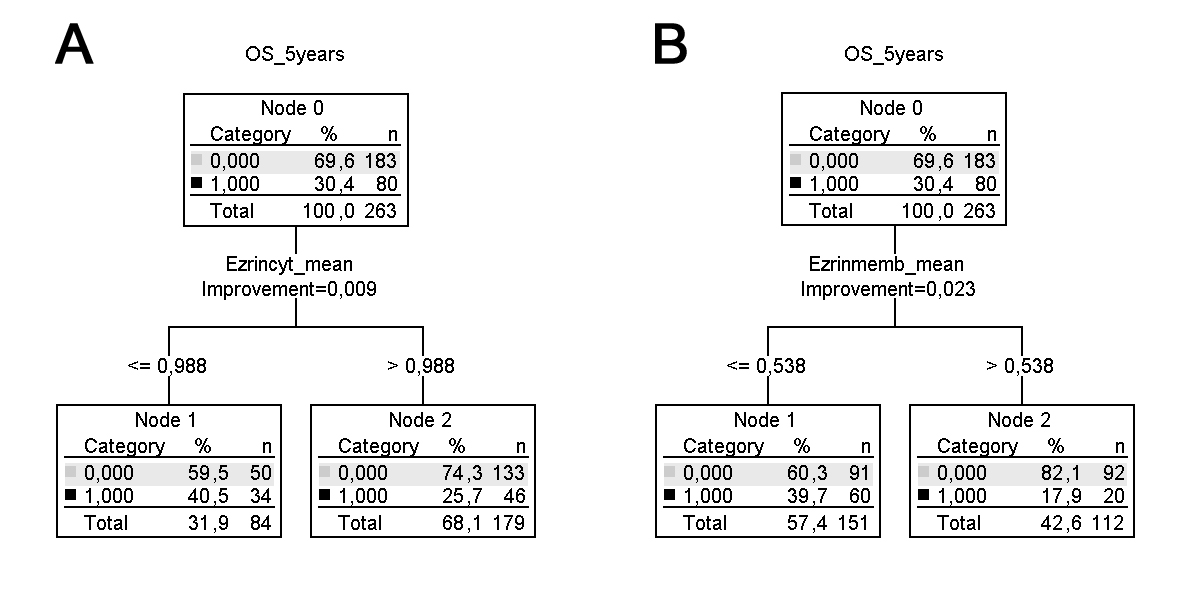

Supplement: Additional file 1: — Classification regression tree analysis for selection of prognostic cutoffs. (A) Cytoplasmic and (B) membranous expression. [file 13000_2014_189_MOESM1_ESM.jpeg]
